# Diversity analyses of bacterial symbionts in four *Sclerodermus* (Hymenoptera: Bethylidae) parasitic wasps, the dominant biological control agents of wood-boring beetles in China

**DOI:** 10.3389/fcimb.2024.1439476

**Published:** 2024-07-25

**Authors:** Kui Kang, Lina Wang, Jun Gong, Yanlong Tang, Ke Wei

**Affiliations:** ^1^ College of Biological and Agricultural Science and Technology, Zunyi Normal University, Zunyi, China; ^2^ Key Laboratory of Forest Protection of National Forestry and Grassland Administration, Ecology and Nature Conservation Institute, Chinese Academy of Forestry, Beijing, China

**Keywords:** *Sclerodermus*, bacterial symbionts, 16S ribosomal RNA amplification sequencing, microbial diversity, *Wolbachia*

## Abstract

**Objective:**

*Sclerodermus* wasps are important biocontrol agents of a class of wood borers. Bacterial symbionts influence the ecology and biology of their hosts in a variety of ways, including the formation of life-long beneficial or detrimental parasitic infections. However, only a few studies have explored the species and content of the symbionts in the *Sclerodermus* species.

**Methods:**

Here, a high-throughput sequencing study of the V3-V4 region of the 16S ribosomal RNA gene revealed a high level of microbial variety in four *Sclerodermus* waps, and their diversities and functions were also predicted

**Results:**

The three most prevalent phyla of microorganisms in the sample were Firmicutes, Bacteroides, and Proteus. The KEEG pathways prediction results indicated that the three pathways with the highest relative abundances in the *S*. *sichuanensis* species were translation, membrane transport, and nucleotide metabolism. These pathways differed from those observed in *S*. *guani*, *S*. *pupariae*, and *S*. *alternatusi*, which exhibited carbohydrate metabolism, membrane transport, and amino acid metabolism, respectively. Bacteroides were found to be abundant in several species, whereas *Wolbachia* was the most abundant among *S. sichuanensis*, with a significant negative correlation between temperature and carriage rate.

**Conclusions:**

These results offer insights into the microbial communities associated with the bethylid wasps, which is crucial for understanding how to increase the reproductive capacity of wasps, enhance their parasitic effects, and lower cost in biocontrol.

## 1 Introduction

Bacterial symbionts have been observed to be long-standing partners of insects, with the capacity to aid their hosts in adapting to a multitude of environmental challenges (Dale and Moran, 2006; Brownlie and Johnson, 2009). The mutually beneficial symbiotic relationship between symbiotic bacteria and insect hosts has significant implications for host biology. Primary bacterial symbionts provide most insects with essential nutrients that they would not obtain from their diets. In contrast secondary bacterial symbionts affect the biological processes of hosts in terms of development, reproduction, and fitness (Kikuchi et al., 2009; Ferrari and Vavre, 2011). Some symbiotic bacteria are transmitted vertically through the infection of reproductive stem cells, blastocyst embryos, or ovarian oocytes in host insect larvae or younger adult females (Braendle et al., 2003; Swiatoniowska et al., 2013; Szklarzewicz and Michalik, 2017). Some host insects obtain symbiotic bacteria from the environment through each generation of individuals, thereby facilitating a horizontal transmission of symbiotic bacteria (Li et al., 2018). The influence of symbiotic bacteria on insect host nutrition, digestion, resistance, and defense responses to natural enemies makes symbiotic bacteria the primary driving force for host colonization and ecological evolution in specific habitats (Douglas, 2015; Damodaram et al., 2016). The fitness of certain *Trichogramma* spp., including *T. delon, T. pretiosum, and T. cordubensis*, can be significantly reduced by infection of *Wolbachia* (Louis, 1993; Silva, 1999; Silva et al., 2000).

The majority of microorganisms that inhabit the gut of insects originate from the food they consume and the external environment. Furthermore, the gut microorganisms of different insect individuals undergo dynamic changes in response to various factors, including insect feeding habits, age, and external environmental conditions. For example, the gut microbial community of *Spodoptera frugiperda* feeding on oilseed rape was significantly higher than that in individuals feeding on wild oats (Lv et al., 2021). The gut microbiotas of the domesticated silkworm greatly differ between early (L1 and L2) and late (L3 and thereafter) instars, and also differ from those of wild mulberry-feeding lepidopterans (Chen et al., 2018). *Wolbachia* are not transmitted to the next generation when immature stages experience cyclical temperatures of 26°C - 37°C during development (Ross et al., 2016). Furthermore, external pathogen infestation represents a significant contributing factor to host gut microbial variation. In *S. exigua*, there is different microbiota composition in field insects carrying a natural viral (Martinez-Solis et al., 2020). In addition, the pathogenic fungus *Beauveria bassiana* infection causes overgrowth and translocation of the opportunistic pathogen *Serratia marcescens* from the gut to the hemocoel, thereby promoting mosquito mortality (Wei et al., 2017).

The bethylid wasps in genus of *Sclerodermus* (Hymenoptera: Bethylidae), such as *Sclerodermus guani* Xiao et Wu*, S. sichuanensis* Xiao*, S. pupariae* Yang et Yao, and *S. alternatusi* Yang are parasitoids that parasitize longhorned beetles and buprestid beetles (Yang et al., 2014; Zhuo et al., 2016), and those four *Sclerodermus* are the most widely used as natural enemies in China. Since the discovery and recording of *Sclerodermus*, great knowledge has been acquired of many areas such as biological characteristics, parasitic habits, and biological control applications. Genetic improvement of parasitic natural enemies by symbionts can markedly enhance their efficacy as natural enemies, thereby reducing the number of natural enemies required for release into the field. which plays a pivotal role in the pest biological control by natural enemies in the field (Allen et al., 2007; Wei et al., 2017). A previous prediction indicated that *S. guani* is infected with *Wolbachia* at high densities, which influences lower quantity of male offspring through cytoplasmic incompatibility (CI) (Zhou and Li, 2014). However, the species and content of the symbionts in these four *Sclerodermus* species remain unclear. In this study, we used 16S rRNA sequence analysis to investigate the species and content of symbionts in these four *Sclerodermus* species. The aim of this study was to provide further details on the global phylogenetic diversity of bacterial symbionts of the four *Sclerodermus* species. This may provide further insights into the functions of these symbionts in *Sclerodermus* biology and found symbiotic bacteria whose functions have been investigated in other insects, especially those that have an effect on the fertility, so as to lay the foundation for subsequent studies.

## 2 Materials and methods

### 2.1 Insects

Four *Sclerodermus* species were used in this study, including *S. guani, S. sichuanensis, S. pupariae*, and *S. alternatusi*. The four species of parasitoids were reared from the same host *Thyestilla gebleri* Faldermann. The larvae of *T. gebleri* were collected from the roots of infested *Abutilon theophrasti* Medicus from Dagang District (38°56’N, 117°29’E), Tianjin City, China. A wild-collected female parasitoid was inoculated into *T. gebleri* larva in a small glass vial (diameter: 1 cm; length: 5 cm). The parasitoid colonies were established in environmental chambers under the standard conditions of temperature 25 ± 1°C, 55–65% relative humidity and 10 h light: 14 h dark regime. The laboratory colonies of the four *Sclerodermus* species were cultured in four independent containers.

Twenty-four *T. gebleri* mature larvae (weight, 200.0–220.0 mg; weighed using an analytical balance having sensitivity of 0.1 mg) were selected for rearing the parasitoids and were randomly and equally divided into four groups. Six mated winged one-week-old females of each *Sclerodermus* specie were randomly selected from the laboratory cultures. Each rearing vial contained one host larva and one female parasitoid. All parasitoids were inoculated on the same day to minimize the potential influence of temporal variability on the experimental results. One newly emerged female adult parasitoids of the next generation in each rearing vial were selected to DNA extraction and detect the bacterial symbionts, and each parasitoid species had six biological replicates

### 2.2 DNA extraction

Before DNA extraction, the insects were immersed in 75% ethanol for 30 s to wash off the bacteria on the surface, then rinsed with ddH_2_O three times. The whole insects were then pre-processed according to the 16S rRNA Earth Microbiome Protocol (Earthmicrobiome.org). Then, gDNA was extracted from samples using the QIAamp^®^ Fast DNA Stool Mini Kit (QIAGEN, Hilden North Rhine-Westphalia, Germany) according to the manufacturer’s instructions. The combined duplicate DNA extracts were purified using a DNA gel extraction kit (Axygen Biosciences, Union City, CA, USA). A BioTek Epoch Microplate Spectrophotometer (Agilent Technologies, Santa Clara, CA, USA) was used to quantify each sample (Chen et al., 2018).

### 2.3 Library construction and 16s rRNA sequencing

The extracted DNA samples were used as templates for PCR amplification targeting the V3-V4 region of 16s rRNA. The forward primer was 341F (-5-CCTACGGGNGGCWGCAG-3-), and the reverse primer was 806R (-5-GGACTACHVGGGTATCTAAT-3-). Each PCR reaction volume was 30 μL, containing 15 μL of 2×Taq Master Mix, 20–30 ng of template DNA, 1 μL of forward/reverse primer. The indexed adapters were attached to the ends of the amplicons to generate sequencing libraries. The amplification procedure was as follows: 94°C for 4 min; 94°C for 30 s, 54°C, 30 s; and 72°C, 1 min for 40 cycles. PCR products were stored at -20°C immediately after the end of the reaction. Using the Illumina HiSeq 2500 platform (Illumina, San Diego, CA, USA) at Biomarker Technologies Co., Ltd. (Beijing, China), the PCR products were used to construct sequencing libraries according to standard protocols.

### 2.4 Raw data processing

After rarefying according to the sequencing depth with a custom script, the paired-end sequence data obtained by HiSeq sequencing were merged into a sequence of tags based on the overlapping relationship. The quality of the reads and the merged effect were quality-controlled and filtered. FLASH v1.2.11 software was used to stitch the reads of each sample through overlap and obtain the original tags, and Trimmomatic v0.33 software was used to filter the spliced raw tags to obtain clean tags. Finally, effective tags were obtained by removing the chimeric sequence using UCHIME v4.2 software.

### 2.5 Taxonomic analysis

Operational taxonomic units (OTUs) were clustered using UPARSE (version 9.2.64) software at a similarity level of 97% (Edgar, 2013). Taxonomic annotation of the OTUs was performed using the Greengene database (version gg_13_5). A Venn graph shows the number of OTUs that are common and unique among samples, and visually shows the overlap of OTUs among samples. By combining with the species represented by the OTUs, it was possible to identify common microorganisms among different species. Venn diagrams for each classification level were drawn using the R software VennDiagram.

### 2.6 Bacterial diversity analysis and function prediction

Alpha diversity reflects the richness and diversity of a single sample with four measurement indicators: Chao1, abundance-based coverage estimator (ACE), Shannon, Simpson, observed species (Sob), and Good’s coverage. The Chao1 and ACE indices were used to evaluate species abundance, while the Simpson and Shannon indices were used to evaluate species diversity and were influenced by the abundance of species in the sample community and community evenness; Sob indicated the type of OTU that could be detected, and Good’s coverage was used to reflect the low abundance OTU coverage of the sample. The alpha diversity index of the samples was evaluated using Qiime software (version 1.9.1). The module “cmdscale” in R software was used to perform principal coordinate analysis. Finally, PICRUSt (version 2.1.4) software was used to annotate the KEGG pathway function of the community in combination with the integrated microbial gene (img) database, and the abundance information of each pathway and KO ID were enumerated.

### 2.7 Wolbachia detection

In order to explore the effects of high temperature on the *Wolbachia* infection of the *S. sichuanensis*, the parasitoids were reared under three temperature gradients (27, 30 and 33°C). The first generation used the previously described, and four consecutive generations were reared in those different temperature treatments (25°C as control). Then 40 mated winged one-week-old females from each temperature treatment were randomly selected for detection. DNA was extracted from a single parasitoid using a previously described method and purified for use as a template for PCR amplification. The wsp-specific primers were wsp81F (5′-TGGTCCAATAAGTGATGAAGAAAC-3′) and wsp691R (5′-AAAAATTAAACGCTACTCCA-3′) (Zhou et al., 1998). PCR reaction was added to 0.5 μL DNA, 10 μL of 2×plus Tap HiFi PCR mix (MIKX, Guangzhou, China), 0.5 μL F/R primer (10 µM) and 8.5 μL ddH_2_O, under PCR conditions of 3 min at 95°C; 25 s at 94°C, 25 s at 58°C and 30 s at 72°C with 40 cycles; 5 min at 72°C. PCR products were detected by 1% agarose gel and purified using a Gel Extraction Kit (OMEGA Bio-Tek, USA), then sequenced by Sangon Biotech (Shanghai) Co., Ltd. (Shanghai, China). The sequencing results using tBLASTn searches against the NCBI data-base to confirm that the cloning sequences belong to *Wolbachia*, and counted the number of parasitoid individuals which containing *Wolbachia* under different temperature conditions to calculate infection frequency. Each temperature treatment had three replicates.

### 2.8 Statistical analysis

All statistical analyses were performed using IBM SPSS Statistics 21 (IBM, Armonk, NY, USA). Differences compared among different species or different temperatures were analyzed using one-way analysis of variance (ANOVA), followed by Least-Significant Difference (LSD) test. The results were considered statistically significant if the *p <*0.05.

## 3 Results

### 3.1 Overview of the sequencing data

**Figure 1:**
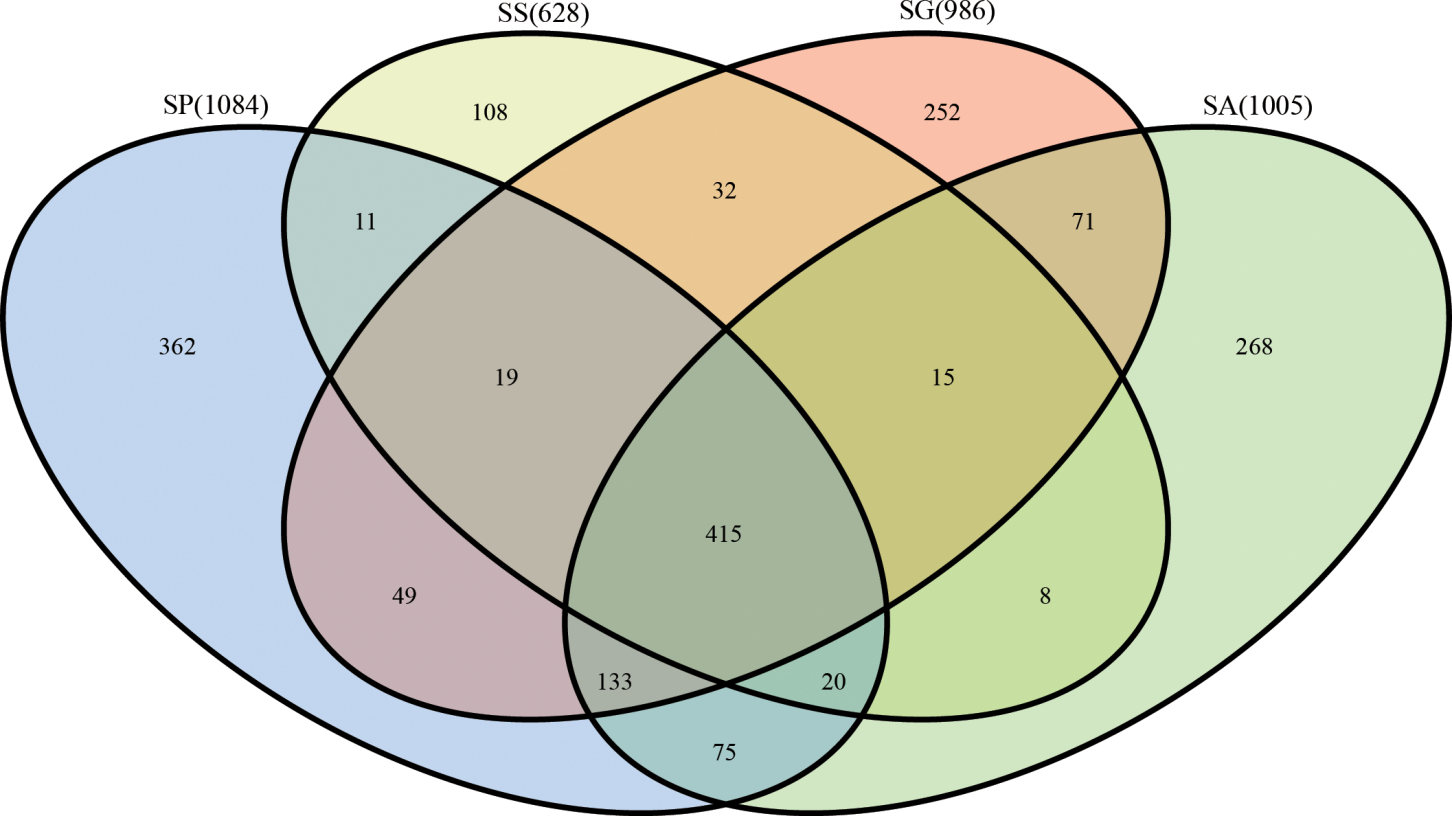
Comparative analysis of OTUs in four species of *Sclerodermus*. SG, *Sclerodermus guani* Xiao et Wu; SS, *Sclerodermus sichuanensis* Xiao; SP, *Sclerodermus pupariae* Yang et Yao; SA, *Sclerodermus alternatusi* Yang.

After 16S rRNA sequencing of four different species of the bethylid wasps *S. guani* (SG)*, S. sichuanensis* (SS)*, S. pupariae* (SP)*, S. alternatusi* (SA), the values of low abundance OTUs coverage of sequenced samples were greater than 0.99 (**Table 1**), which means that the accuracy and annotation coverage of the sequencing results were high and can meet the requirements of subsequent analysis. In total, 344234 reads (each with an average length of 454 bp) were obtained. After quality control and screening, 299249 reads were obtained for subsequent analysis. The total proportion of effective tags was 86.93%, and the proportion of each group of samples was greater than 84% (**Table 1**). Based on 97% sequence similarity, the effective tags were clustered, and the OTUs with sequence number <0.005% were filtered. Finally, 1838 OTUs were clustered in all samples. A comparative analysis of OTUs in the four species showed that 415 OTUs were clustered in four species: 1084 in SP, 362 unique; 628 in SS, 108 unique; 986 in SG, 252 unique; and 1005 in SA, 268 unique (**Figure 1**).

**Table 1:** Sequencing and quality assessment of different species of parasitoids.

Group	Raw PE	Effective Tags	Effective Ratio (%)	Goods coverage
SP	92268	77683	84.05	0.995
SS	74848	64693	86.40	0.994
SG	86082	75726	87.67	0.994
SA	91036	81147	89.38	0.995

SG, *Sclerodermus guani* Xiao et Wu; SS, *Sclerodermus sichuanensis* Xiao; SP, *Sclerodermus pupariae* Yang et Yao; SA, *Sclerodermus alternatusi* Yang.

### 3.2 Species and differences in abundance of symbiotic bacteria

Based on the annotation results of the OTUs, symbiotic bacterial communities in different species of *Sclerodermus* were analyzed. The symbiotic bacterial communities were annotated as 26 phyla, 55 classes, 111 orders, 170 families, 312 genera, 152 species. The number of symbiotic bacteria identified to species in SS was relatively low, with a total of 75 species annotated. The numbers of different taxonomic elements in the other three species were found to be similar (**Table 2**).

**Table 2:** Taxonomic information of bacteria in different species of Parasitoids.

Group	Number of different taxonomic categories					
	Phylum	Class	Order	Family	Genus	Species
SP	21	43	82	119	215	110
SS	17	27	58	82	145	75
SG	20	38	75	105	190	100
SA	22	41	75	111	204	99
Total	26	55	111	170	312	152

SG, *Sclerodermus guani* Xiao et Wu; SS, *Sclerodermus sichuanensis* Xiao; SP, *Sclerodermus pupariae* Yang et Yao; SA, *Sclerodermus alternatusi* Yang.

**Figure 2:**
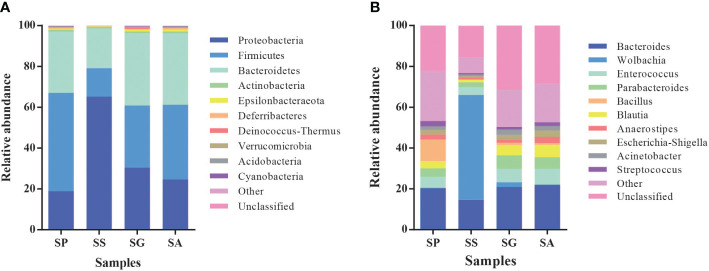
Relative abundances of microbiota phyla **(A)** and genera **(B)** in different species of Parasitoids. SG, *Sclerodermus guani* Xiao et Wu; SS, *Sclerodermus sichuanensis* Xiao; SP, *Sclerodermus pupariae* Yang et Yao; SA, *Sclerodermus alternatusi* Yang.

At the phylum level (**Figure 2A**), the symbionts of the four species were predominantly Firmicutes, Bacteroidetes, and Proteobacteria. However, the relative abundance of colonies exhibited notable variation among different species. In SP, the abundance of the main symbiotic bacteria was found to be as follows: 48.3% Firmicutes, 29.8% Bacteroidetes, and 18.9% Proteobacteria. In SS, the predominant symbiotic bacteria were identified as belonging to the Proteobacteria (65.2%), Bacteroidetes (19.5%), and Firmicutes (14.0%). In SG, the most prevalent symbionts were classified as Bacteroidetes (35.2%), Firmicutes (30.5%), and Proteobacteria (30.4%). In SA, the abundance of the main symbionts ranked as Firmicutes (36.6%), Bacteroidetes (35.1%), and Proteobacteria (24.6%).

At the genera level (**Figure 2B**; **Supplementary Figure S1**), the compositions of the symbiotic bacterial communities of the four wasp species were significantly different (**Supplementary Table S1**). In SP, *Bacteroides* (20.4%) and *Enterococcus* (5.4%) were the most prevalent symbionts, exhibiting high abundance. The predominant symbiotic bacteria in SS were identified as *Wolbachia* (51.4%), *Bacteroides* (14.6%), and *Enterococcus* (3.8%). In SG, *Bacteroides* (20.9%), *Parabacteroides*, (6.6%) and *Enterococcus* (6.5%) were the main symbionts with high abundance. In SA, *Bacteroides* (22.0%), *Enterococcus* (7.8%), *Blautia* (6.0%), and *Parabacteroides* (5.7%) were the main symbionts with high abundance.

### 3.3 Diversity and abundance differences of symbiotic bacteria in *Sclerodermus*

**Figure 3:**
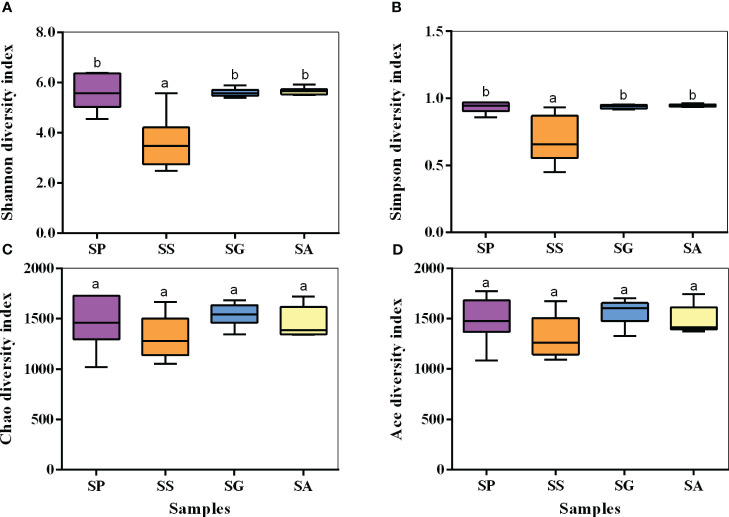
Alpha diversity, Shannon index **(A)**, Simpson index **(B)**, Chao1 index **(C)**, and Ace index **(D)**, for the bacterial communities. SG, *Sclerodermus guani* Xiao et Wu; SS, *Sclerodermus sichuanensis* Xiao; SP, *Sclerodermus pupariae* Yang et Yao; SA, *Sclerodermus alternatusi* Yang. Different letters over the points indicate a significant difference using Least-Significant Difference (LSD) test (*P* < 0.05).

The alpha diversity index was used to analyze the diversity and richness of each sample (**Figure 3**). The Chao1/ACE index mainly shows the species richness of a sample. The larger the index, the higher is the richness. The Simpson-Shannon index comprehensively reflects the evenness of species. The larger the index, the higher is the evenness. Group SS had the smallest Shannon and Simpson indices, which were significantly lower than those of the other three species (Shannon: *F*=14.323, *df*=3,23, *p*<0.001; Simpson: *F*=11.501, *df*=3,22, *p*<0.001). There were no significant differences in the Chao1 (*F*=1.369, *df*=3,23, *p*=0.281) and ACE (*F*=1.912, *df*=3,23, *p*=0.160) indices among the four species.

### 3.4 Community function prediction

According to the species annotation and abundance information of the OTUs, Picrust software was used to annotate the function of the KEGG pathway and determine the abundance information of each pathway. The KEGG pathways with higher relative abundance predicted by the symbionts of different species of the bethylid wasps were the same (**Table 3**). However, the three pathways with the highest relative abundances in the SS species were translation, membrane transport, and nucleotide metabolism, which differed from the carbohydrate metabolism, membrane transport, and amino acid metabolism observed in the other three species.

**Table 3:** The relative abundance of pathway.

KEGG Pathway	Relative abundance			
	SP	SS	SG	SA
Carbohydrate Metabolism	0.1494 ± 0.0023	0.0890 ± 0.0047	0.1418 ± 0.0075	0.1522 ± 0.0006
Membrane Transport	0.1175 ± 0.0020	0.1035 ± 0.0015	0.1181 ± 0.0020	0.1201 ± 0.0017
Amino Acid Metabolism	0.1069 ± 0.0014	0.0823 ± 0.0017	0.1003 ± 0.0026	0.1036 ± 0.0003
Signal Transduction	0.0702 ± 0.0015	0.0272 ± 0.0029	0.0634 ± 0.0055	0.0690 ± 0.0011
Metabolism of Cofactors and Vitamins	0.0676 ± 0.0004	0.0809 ± 0.0010	0.0687 ± 0.0015	0.0660 ± 0.0004
Energy Metabolism	0.0625 ± 0.0006	0.0893 ± 0.0022	0.0657 ± 0.0029	0.0606 ± 0.0006
Nucleotide Metabolism	0.0625 ± 0.0012	0.0928 ± 0.0022	0.0667 ± 0.0039	0.0633 ± 0.0007
Translation	0.055 ± 0.0010	0.1167 ± 0.0045	0.0643 ± 0.0076	0.0562 ± 0.0008
Replication and Repair	0.0519 ± 0.0010	0.0824 ± 0.0022	0.0562 ± 0.0039	0.0527 ± 0.0006
Glycan Biosynthesis and Metabolism	0.0361 ± 0.0014	0.0268 ± 0.0008	0.0357 ± 0.0013	0.0379 ± 0.0003
Folding, Sorting and Degradation	0.0263 ± 0.0003	0.0425 ± 0.0012	0.0286 ± 0.0019	0.0263 ± 0.0001
Lipid Metabolism	0.0337 ± 0.0004	0.0291 ± 0.0004	0.0331 ± 0.0006	0.0337 ± 0.0001

SG, *Sclerodermus guani* Xiao et Wu; SS, *Sclerodermus sichuanensis* Xiao; SP, *Sclerodermus pupariae* Yang et Yao; SA, *Sclerodermus alternatusi* Yang.

### 3.5 *Wolbachia* infection frequency of *S. sichuanensis* at different temperatures

**Figure 4:**
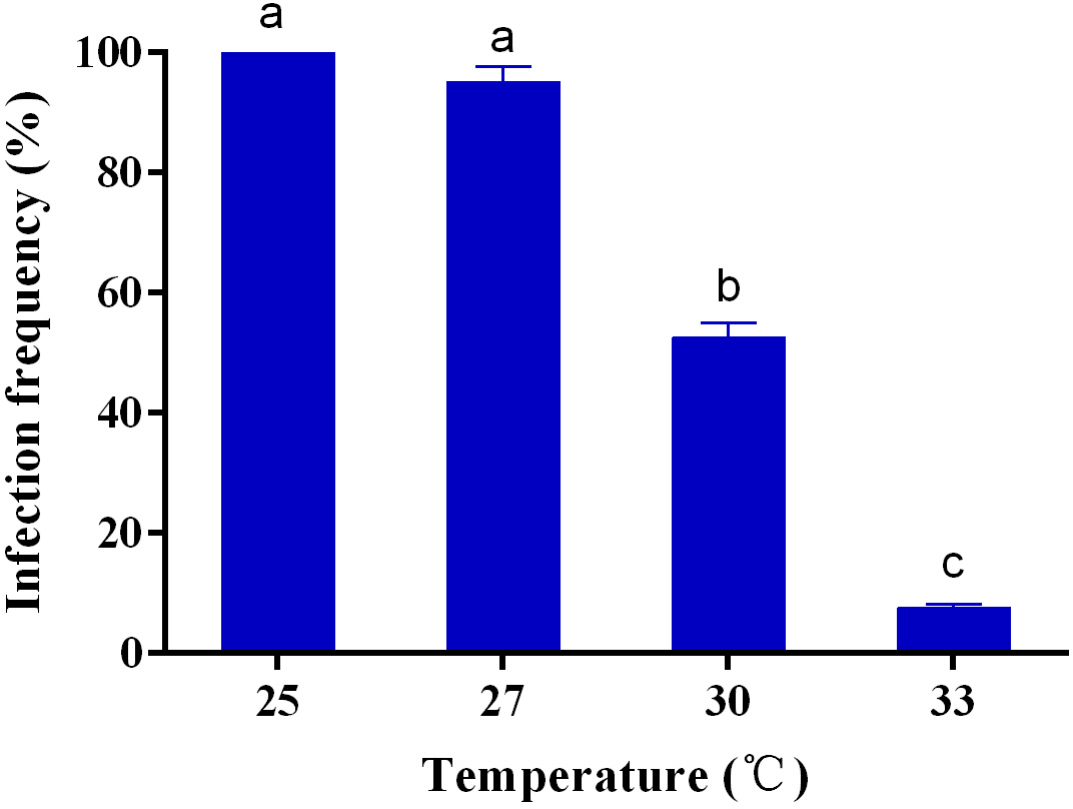
*Wolbachia* infection frequency of *S. sichuanensis* at different temperatures. Data are shown as the means ± SE of three replicates. Different letters on the bars indicate a significant difference using Least-Significant Difference (LSD) test (*P* < 0.05).

There was a significant difference (*F*=400.244, *df*=3,11, *p*<0.001) in the infection frequency of *Wolbachia* after 4 generations of rearing at different temperatures, with a significant negative correlation between temperature and infection frequency (**Figure 4**). Infection frequency of *Wolbachia* was still 95.0% after 4 generations of rearing at 27°C but decreased to 52.5% when the temperature was raised to 30°C. After 4 generations of rearing at 33°C, the infection frequency of *Wolbachia* was only 7.5% (**Figure 4**).

## 4 Discussion

In this study, the Illumina MiSeq high-throughput sequencing method of the 16S rRNA V3 + V4 hypervariable region was used to systematically analyze the microbial diversity in the four species of *Sclerodermus*. The numbers of symbiotic bacteria in these *Sclerodermus* parasitoids were not significantly different; however, the community composition was significantly different, especially in *S. sichuanensis*. The most abundant genera were *Wolbachia*, which differed significantly from the other three species. Previous studies have shown that symbiotic bacteria can induce host parthenogenesis in many parasitic wasps. After reducing the titer of *Wolbachia* in *Encarsia formosa* by antibiotic treatment, the parasitic wasp that produces female parthenogenesis begins to produce male offspring (Wang et al., 2017). *Cardinium* can cause parthenogenesis in the aphid wasp *E. pergandiella* (Kenyon and Hunter, 2007). Owing to the involvement of *Wolbachia*, two sets of chromosomes fail to separate in the egg cells of some insects at the late stage of the first mitosis, resulting in two sets of completely identical chromosomes in unfertilized egg cells (Stouthamer and Kazmer, 1994).

In addition, the *Enterococcus* content was found to be high in several parasitic wasps, and *Enterococcus* and *Enterobacter* were the most common dominant bacteria in the intestinal tracts of lepidopteran insects (Broderick et al., 2004; Teh et al., 2016). *Enterobacter* spp. is abundant in eggs and pupae and can increase the fitness of pupae and adults (Augustinos et al., 2015), which has been shown to have high metabolic adaptability in both the egg and larval stages of insects. A large number of *Actinobacteria* was also detected in the 1st instar larvae and male adults, similar to the microbial community composition of *Chrysoperla sinica* at different developmental stage (Zhao et al., 2019). Members of the bacterial phylum Actinobacteria are especially prevalent as defensive symbionts due to their ecological and physiological prerequisites, including the ability to utilize a diverse range of nutritional resources and a remarkable versatility in producing secondary metabolites with antibiotic properties (Kaltenpoth, 2009). In scarab beetles of the genus *Pachnoda*, a number of bacterial strains with hemicellulolytic capabilities were isolated from the hindgut, including *Promicromonospora pachnodae*, an actinobacterial species capable of producing a range of xylanases and endoglucanases - two enzyme families involved in (hemi) cellulose degradation (Andert et al., 2010). The dominant bacteria in insects vary from species to species, but the resident bacteria belonging to insects, Proteobacteria, Bacteroidetes, Actinobacteria, and Firmicutes, jointly dominate the insect microbial community and play important roles in insect physiology and metabolism. *Amylostereum areolatum*, belonging to Proteobacteria putatively encoding CAZymes, has complementary functions for degrading woody substrates and that such degradation may assist in nutrient acquisition by *Sirex noctilio* (Nakamatsu and Tanaka, 2004; Engel et al., 2012).

Parasitic wasps regulate host lipid metabolism, host triglyceride and fatty acid content (Kaeslin et al., 2005), and host fatty acid composition (Thompson and Barlow, 1974), providing a favorable environment for their offspring larvae, but also withstanding host defense. Previous studies have found that *Enterococcus* sp. as a symbiotic bacterium of *Plutella xylostella* can effectively regulate the immune system of host insects and improve their resistance to poisoned cicadas (Xia et al., 2018). In addition, both *Enterobacter* and *Serratia* can degrade poisoned cockroaches and stimulate the immune function of the host *P. xylostella*, thereby increasing the resistance of *P. xylostella* (Daisley et al., 2018). *Serratia* in aphids can significantly improve the host survival ability at high temperatures (Russell and Moran, 2006). The intestinal symbiotic bacteria of *Bactrocera dorsalis* can prolong the time that *B. dorsalis* can tolerate temperature stress, and the types of symbiotic bacteria in *B. dorsalis* vary with temperature (Ayyasamy et al., 2021).

The results of the present study showed that the content of *Wolbachia* in *S. sichuanensis* was higher than that in other species, and the infection frequency of *Wolbachia* was decreases with the increase of rearing temperature. *Wolbachia* is the most prevalent intracellular facultative symbiotic bacterium in arthropods and is stably colonized in the majority of arthropods through cytoplasmic inheritance (Hoffmann, 2020). Stresses such as temperature are well-known environmental variables that decrease *Wolbachia* densities and expression of reproductive parasitism across host species (Snook et al., 2000). In *Nasonia vitripennis*, heat treatment at 30°C reduce *Wolbachia* densities by as much as 74% relative to wasps reared at 25°C (Bordenstein and Bordenstein, 2011). In recent years, the functions of *Wolbachia* have been further elucidated, including its role in mediating host resistance to viruses, influencing behavior, memory, olfaction, intestinal microbial diversity, and temperature preference (Moreira et al., 2009; Bi and Wang, 2020; Hague et al., 2020). Additionally, *Wolbachia* can confer stress resistance to the host, markedly enhancing the host’s survival capacity in the presence of insecticides and heavy metal stress (Burdina et al., 2021; Cai et al., 2021). This suggests that *Wolbachia* can also enhance the host’s fitness under stressful conditions while influencing its behavior and reproduction. The previous study indicated that *Wolbachia* in *S. guani* was identical with that of the strain in supergroup A, and it was predicted that it should be a CI-inducing strain (Zhou and Li, 2014). In the present study, the prediction of community function was relatively concentrated on carbohydrate metabolism in the species of SP, SG, and SA. Parasitic wasps disrupt the host’s normal metabolic processes, resulting in notable alterations in metabolite concentrations. These changes influence the host’s nutrient composition, enabling the parasitic wasps to more effectively utilize nutritional resources (Pennacchio et al., 1994). Trehalose represents the primary component of blood sugar in host body fluids. In insects that feed on high-sugar foods, symbiotic bacteria utilize sugars to synthesize acetate and other products that are required by the host (Lievens et al., 2015). The intestinal symbiotic bacteria of *Drosophila melanogaster*, which feed on high-sugar rotten fruits, metabolize polysaccharides to produce three- to five-carbon alcohols, aldehydes, acids, and esters (Becher et al., 2012). The *in vitro* interaction of various symbiotic bacteria can enhance the yield and diversity of digestive products; *Drosophila* also prefers the digestive products of various symbiotic bacteria (Pennacchio et al., 1994). *Acetobacter pomorum* uses lactic acid produced by *Lactobacillus plantarum* to synthesize acetoin, whereas *A. malorum* uses ethanol produced by *Saccharomyces cerevisiae* to synthesize acetic acid (Farine et al., 2017; Fischer et al., 2017). The primary predictive function of the community in *S. sichuanensis* is translation, which may affect the regulation of nutrient metabolism in the host. For instance, in *D. melanogaster*, the intracellular symbiont *Wolbachia* modulates the expression of the sirt-4 gene, which in turn influences the expression of the host glutamate dehydrogenase (a key enzyme in glucose metabolism) and host carbohydrate metabolism (Dutra et al., 2020). Additionally, intestinal symbiotic bacteria can also use their own metabolites, such as short-chain fatty acids, as a means of communication with their hosts (Koh et al., 2016).

The present study found a substantial diversity of bacterial symbionts among four *Sclerodermus* species. The findings of this study provide a foundation for further investigation into the function of the intestinal endophytic flora in *Sclerodermus* parasitoids, with a particular focus on biological characteristics such as reproductive regulation and host search. Furthermore, these findings will enhance our understanding of the symbiotic relationship between symbiotic bacteria and parasitic wasps, and offer novel insights and methodologies for the population dynamics of the *Sclerodermus* parasitoids.

## Data availability statement

The datasets presented in this study can be found in online repositories. The data can be found in NCBI Bioproject repository, [PRJNA953092].

## Ethics statement

The manuscript presents research on animals that do not require ethical approval for their study.

## Author contributions

KK: Conceptualization, Data curation, Formal analysis, Investigation, Methodology, Writing – original draft, Writing – review & editing. LW: Data curation, Methodology, Writing – original draft. JG: Data curation, Methodology, Writing – original draft. YT: Conceptualization, Funding acquisition, Data curation, Formal analysis, Writing – original draft, Writing – review & editing. KW: Conceptualization, Methodology, Formal analysis, Funding acquisition, Writing – original draft, Writing – review & editing.

## References

[B1] Allen J. M. Reed D. L. Perotti M. A. Braig H. R. (2007). Evolutionary relationships of “*Candidatus* Riesia spp.,” endosymbiotic enterobacteriaceae living within hematophagous primate lice. Appl. Environ. Microbiol. 73, 1659–1664. doi: 10.1128/AEM.01877-06 17220259 PMC1828778

[B2] Andert J. Marten A. Brandl R. Andreas B. (2010). Inter-and intraspecific comparison of the bacterial assemblages in the hindgut of humivorous scarab beetle larvae (*Pachnoda* spp.). FEMS Microbiol. Ecol. 74, 439–449. doi: 10.1111/fem.2010.74.issue-2 20738398

[B3] Augustinos A. A. Kyritsis. G. A. Papadopoulos N. T. Abd-Alla A. M. M. Cáceres C. Bourtzis K. (2015). Exploitation of the medfly gut microbiota for the enhancement of sterile insect technique: use of Enterobacter sp. in larval diet-based probiotic applications. PloS One 10, 136459. doi: 10.1371/journal.pone.0136459 PMC4556606 26325068

[B4] Ayyasamy A. Kempraj V. Damodaram K. J. P. (2021). Endosymbiotic bacteria aid to overcome temperature induced stress in the oriental fruit fly. Bactrocera dorsalis. Microb. Ecol. 82, 783–792. doi: 10.1007/s00248-021-01682-2 33559710

[B5] Becher P. G. Flick G. Rozpedowska E. Schmidt A. Hagman A. Lebreton S.. (2012). Yeast, not fruit volatiles mediate *Drosophila melanogaster* attraction, oviposition and development. Funct. Ecol. 26, 822–828. doi: 10.1111/j.1365-2435.2012.02006

[B6] Bi J. Wang Y. F. (2020). The effect of the endosymbiont *Wolbachia* on the behavior of insect hosts. Insect Sci. 27, 846–858. doi: 10.1111/1744-7917.12731 31631529 PMC7496987

[B7] Bordenstein S. R. Bordenstein S. R. (2011). Temperature affects the tripartite interactions between bacteriophage WO, *Wolbachia*, and cytoplasmic incompatibility. PloS One 6, e29106. doi: 10.1371/journal.pone.0029106 22194999 PMC3240643

[B8] Braendle C. Miura T. Bickel R. Shingleton A. W. Kambhampati S. Stern D. L. (2003). Developmental origin and evolution of bacteriocytes in the aphid-Buchnera symbiosis. PloS Biol. 1, 70–76. doi: 10.1371/journal.pbio.0000021 PMC212699 14551917

[B9] Broderick N. A. Raffa K. F. Goodman R. M. Handelsman J. (2004). Census of the bacterial community of the gypsy moth larval midgut by using culturing and culture-independent methods. Appl. Environ. Microb. 70, 293–300. doi: 10.1128/AEM.70.1.293-300.2004 PMC321235 14711655

[B10] Brownlie J. C. Johnson K. N. (2009). Symbiont-mediated protection in insect hosts. Trends Microbiol. 17, 348–354. doi: 10.1016/j.tim.2009.05.005 19660955

[B11] Burdina E. V. Bykov R. A. Menshanov P. N. Ilinsky Y. Y. Gruntenko N.Е. (2021). Unique *Wolbachia* strain MelPlus increases heat stress resistance in *Drosophila melanogaster* . Arch. Insect Biochem. Physiol. 106, e21776. doi: 10.1002/arch.21776 33644932

[B12] Cai T. Zhang Y. Liu Y. Deng X. Q. He S. Li J. H.. (2021). *Wolbachia* enhances expression of NLCYP4CEI in *Nilaparvata lugens* in response to imidacloprid stress. Insect Sci. 28, 355–362. doi: 10.1111/1744-7917.12834 32519810

[B13] Chen B. S. Du K. Q. Sun C. Vimalanathan A. Liang X. L. Li Y.. (2018). Gut bacterial and fungal communities of the domesticated silkworm (*Bombyx mori*) and wild mulberry-feeding relatives. ISME J. 12, 2252–2262. doi: 10.1038/s41396-018-0174-1 29895989 PMC6092317

[B14] Daisley B. A. Trinder M. Mcdowell T. W. Collins S. L. Reid G. (2018). Microbiota-mediated modulation of organophosphate insecticide toxicity by species. dependent interactions with *Lactobacilli* in a *Drosophila melanogaster* insect model. Appl. Environ. Microbiol. 84, e02820–e02817. doi: 10.1128/AEM.02820-17 29475860 PMC5930343

[B15] Dale C. Moran N. A. (2006). Molecular interactions between bacterial symbionts and their hosts. Cell 126, 453–465. doi: 10.1016/j.cell.2006.07.014 16901780

[B16] Damodaram K. J. P. Ayyasamy A. Kempraj V. (2016). Commensal bacteria aid mate-selection in the fruit fly, *Bactrocera dorsalis* . Microb. Ecol. 72, 725–729. doi: 10.1007/s00248-016-0819-4 27423980

[B17] Douglas A. E. (2015). Multiorganismal insects: diversity and function of resident microorganisms. Annu. Rev. Entomol. 60, 17–34. doi: 10.1146/annurev-ento-010814-020822 25341109 PMC4465791

[B18] Dutra H. L. C. Deehan M. A. Frydman H. (2020). *Wolbachia* and Sirtuin-4 interaction is associated with alterations in host glucose metabolism and bacterial titer. PloS Pathog. 16, e1008996. doi: 10.1371/journal.ppat.1008996 33048997 PMC7584242

[B19] Edgar R. C. (2013). UPARSE: highly accurate OTU sequences from microbial amplicon reads. Nat. Methods 10 (10), 996–998. doi: 10.1038/nmeth.2604 23955772

[B20] Engel P. Martinson V. G. Moran N. A. (2012). Functional diversity within the simple gut microbiota of the honey bee. Proc. Natl. Acad. Sci. U.S.A. 109, 11002–11007. doi: 10.1073/pnas.1202970109 22711827 PMC3390884

[B21] Farine J. P. Habbachi W. Cortot J. Roche S. Ferveur J. F. (2017). Maternally-transmitted microbiota affects odor emission and preference in *Drosophila* larva. Sci. Rep. 7, 6062. doi: 10.1038/s41598-017-04922-z 28729609 PMC5519639

[B22] Ferrari J. Vavre F. (2011). Bacterial symbionts in insects or the story of communities affecting communities. Philos. Trans. R Soc Lond. B Biol. Sci. 366, 1389–1400. doi: 10.1098/rstb.2010.0226 21444313 PMC3081568

[B23] Fischer C. N. Trautman E. P. Crawford J. M. Stabb E. V. Handelsman J. Broderick N. A. (2017). Metabolite exchange between microbiome members produces compounds that influence *Drosophila* behavior. eLife 6, e18855. doi: 10.7554/eLife.18855 28068220 PMC5222558

[B24] Hague M. T. J. Caldwell C. N. Cooper B. S. (2020). Pervasive effects of *Wolbachia* on host temperature preference. mBio. 11, e01768–e01720. doi: 10.1128/mBio.01768-20 33024036 PMC7542361

[B25] Hoffmann A. (2020). Wolbachia. Curr. Biol. 30, R1113–R1114. doi: 10.1016/j.cub.2020.08.039 33022249

[B26] Kaeslin M. Pfister-Wilhelm R. Molina D. Lanzrein B. (2005). Changes in the haemolymph proteome of *Spodoptera littoralis* induced by the parasitoid *Chelonus inanitus* or its polydnavirus and physiological implications. J. Insect Physiol. 51, 975–988. doi: 10.1016/j.jinsphys.2005.04.012 15936028

[B27] Kaltenpoth M. (2009). Actinobacteria as mutualists: general healthcare for insects? Trends Microbiol. 17, 529–535. doi: 10.1016/j.tim.2009.09.006 19853457

[B28] Kenyon S. G. Hunter M. S. (2007). Manipulation of oviposition choice of the parasitoid wasp, *Encarsia Kenyon Spergandiella*, by the endosymbiotic bacterium *Cardinium* . J. Evol. Bio. 20, 707–716. doi: 10.1111/j.1420-9101.2006.01238.x 17305836

[B29] Kikuchi Y. Hosokawa T. Nikoh N. Meng X. Y. Kamagata Y. Fukatsu T. (2009). Host-symbiont co-speciation and reductive genome evolution in gut symbiotic bacteria of acanthosomatid stinkbugs. BMC Biol. 7, 2. doi: 10.1186/1741-7007-7-2 19146674 PMC2637841

[B30] Koh A. De Vadder F. Kovatcheva-Datchary P. Bäckhed F. (2016). From dietary fiber to host physiology: short-chain fatty acids as key bacterial metabolites. Cell 165, 1332–1345. doi: 10.1016/j.cell.2016.05.041 27259147

[B31] Li Q. Fan J. Sun J. Wang M. Q. Chen J. (2018). Plant-mediated horizontal transmission of *Hamiltonella defensa* in the wheat aphid *Sitobion miscanthi* . J. Agric. Food Chem. 6, 13367–13377. doi: 10.1021/acs.jafc.8b04828 30516997

[B32] Lievens B. Hallsworth J. E. Pozo M. I. Belgacem Z. B. Stevenson A. Willems K. A.. (2015). Microbiology of sugar-rich environments: diversity, ecology and system constraints. Environ. Microbiol. 17, 278–298. doi: 10.1111/1462-2920.12570 25041632

[B33] Louis C. (1993). Research on the origin of unisexuality-thermotherapy curea both Rickettsia and the lytokous parthenogenesis in a *Trichogramma species* (Hymenoptera, Ttrichogrammatidae). CR Acad Sci Ser III Scivie 316, 27–33.

[B34] Lv D. Liu X. Dong Y. Yan Z. Zhang X. Wang P.. (2021). Comparison of gut bacterial communities of fall armyworm (*Spodoptera frugiperda*) reared on different host plants. Int. J. Mol. Sci. 22, 11266. doi: 10.3390/ijms222011266 34681926 PMC8540368

[B35] Martinez-Solis M. Collado M. C. Herrero S. (2020). Influence of diet, sex, and viral infections on the gut microbiota composition of *Spodoplera exigua* caterpillars. Front. Microbiol. 11. doi: 10.3389/fmicb.2020.00753 PMC7218101 32435237

[B36] Moreira L. A. Iturbe-Ormaetxe I. Jeffery J. A. Lu G. Pyke A. T. Hedges L. M.. (2009). *Wolbachia* symbiont in *Aedes aEgypti* limits infection with dengue, chikungunya, and Plasmodium. Cell 139, 1268–1278. doi: 10.1016/j.cell.2009.11.042 20064373

[B37] Nakamatsu Y. Tanaka T. (2004). Correlation between concentration of hemolymph nutrients and amount of fat body consumed in lightly and heavily parasitized hosts (*Pseudaletia separata*). J. Insect Physiol. 50, 135–141. doi: 10.1016/j.jinsphys.2003.10.005 15019514

[B38] Pennacchio F. Vinson S. B. Tremblay E. (1994). Biochemical and developmental alterations of *Heliothis virescens* (F) (Lepidoptera, noctuidae) larvae induced by the endophagous parasitoid *CardioChiles nigriceps* viereck (Hymenoptera, braconidae). Arch. Insect Biochem. Physiol. 26, 161–174. doi: 10.1002/arch.940260211

[B39] Ross P. A. Wiwatanaratanabutr I. Axford J. K. White V. L. Endersby-Harshman N. M. Hoffmann A. A. (2016). *Wolbachia* infections in A*edes aEgypti* differ markedly in their response to cyclical heat stress. PloS Pathog. 13, el006006. doi: 10.1371/journal.ppat.1006006 PMC5215852 28056065

[B40] Russell J. A. Moran N. A. (2006). Costs and benefits of symbiont infection in aphids: variation among symbionts and across temperatures. Proc. Biol. Sci. 273, 603–610. doi: 10.1098/rspb.2005.3348 16537132 PMC1560055

[B41] Silva L. M. M. S. (1999). Identification and evaluation of *Trichogramma Parasiotids* biologicai pest control (The Netherlands: Laboratory of Entomology, Waneningen Uinversity), 55.

[B42] Silva L. M. M. S. Meer M. M. M. Roskam M. M. Hoogenboom A. Gort G. Stouthamer R. (2000). Biological control potential of *Wolbachia*-infected versus un-infected wasps: laboratory and greenhouse evaluation of *Trichogramma cordubensis* and *T. dendrolimi* strains. Biocontrol Sci. Techn. 10, 223–238. doi: 10.1080/09583150050044501

[B43] Snook R. R. Cleland S. Y. Wolfner M. F. Karr T. L. (2000). Offsetting effects of Wolbachia infection and heat shock on sperm production in *Drosophila simulans*: analyses of fecundity, fertility and accessory gland proteins. Genetics 155, 167–178. doi: 10.1093/genetics/155.1.167 10790392 PMC1461085

[B44] Stouthamer R. Kazmer D. J. (1994). Cytogenetics of microbe-associated parthenogenesis and its consequences for gene flow in *Trichogramma* wasps. Heredity 73, 317–327. doi: 10.1038/hdy.1994.139

[B45] Swiatoniowska M. Ogorzalek A. Golas A. Michalik A. Szklarzewicz T. (2013). Ultrastructure, distribution and transovarial transmission of symbiotic microorganisms in *Nysius ericae* and *Nithecus jacobaeae* (Heteroptera: Lygaeidae: Orsillinae). Protoplasma 250, 325–332. doi: 10.1007/s00709-012-0416-4 22588432 PMC3557392

[B46] Szklarzewicz T. Michalik A. (2017). Transovarial transmission of symbionts in insects. Results Probl. Cell. Differ. 63, 43–467. doi: 10.1007/978-3-319-60855-6_3 28779313

[B47] Teh B. S. Apel J. Shao Y. Boland W. (2016). Colonization of the intestinal tract of the polyphagous pest *Spodoptera littoralis* with the GFP-tagged indigenous gut bacterium *Enterococcus mundtii* . Front. Microbiol. 7. doi: 10.3389/fmicb.2016.00928 PMC4906056 27379058

[B48] Thompson S. N. Barlow J. S. (1974). The fatty acid composition of parasitic Hymenoptera and its possible biological significance. Ann. Entomol. Soc Am. 67, 627–632. doi: 10.1093/aesa/67.4.627

[B49] Wang X. X. Qi L. D. Jiang R. Du Y. Z. Li Y. X. (2017). Incomplete removal of *Wolbachia* with tetracycline has two-edged reproductive effects in the thelytokous wasp *Encarsia formosa* (Hymenoptera Aphelinidae). Sci. Rep. 7, 44014. doi: 10.1038/srep44014 28266601 PMC5339822

[B50] Wei G. Lai Y. Wang G. Chen H. Li F. Wang S. (2017). Insect pathogenic fungus interacts with the gut microbiota to accelerate mosquito mortality. Proc. Natl. Acad. Sci. U.S.A. 114, 5994–5999. doi: 10.1073/pnas.1703546114 28533370 PMC5468619

[B51] Xia X. Sun B. Gurr G. M. Vasseur L. Xue M. You M. (2018). Gut microbiota mediate insecticide resistance in the diamondback moth, *Plutella xylostella* (L.). Front. Microbiol. 9. doi: 10.3389/fmicb.2018.00025 PMC5787075 29410659

[B52] Yang Z. Q. Wang X. Y. Zhang Y. N. (2014). Recent advances in biological control researches on main native and invasive forest pests in China. Biol. Control 68, 117–128. doi: 10.1016/j.biocontrol.2013.06.010

[B53] Zhao C. Zhao H. Zhang S. Luo J. Zhu X. Wang L.. (2019). The developmental stage symbionts of the pea aphid-feeding *Chrysoperla sinica* (Tjeder). Front. Microbiol. 10. doi: 10.3389/fmicb.2019.02454 PMC6839393 31736900

[B54] Zhou W. Rousset F. O’Neil S. (1998). Phylogeny and PCR-based classification of *Wolbachia* strains using *wsp* gene sequences. Proc. Biol. Sci. 265, 509–515. doi: 10.1098/rspb.1998.0324 9569669 PMC1688917

[B55] Zhou Y. Li Z. X. (2014). Bidirectional cytoplasmic incompatibility induced by cross-order transfection of *Wolbachia*: implications for control of the host population. Microb. Ecol. 68, 463–471. doi: 10.1007/s00248-014-0425-2 24787986

[B56] Zhuo Z. H. Yang W. Xu D. P. Yang C. P. Yang H. (2016). Effects of *Scleroderma sichuanensis* Xiao (Hymenoptera: Bethylidae) venom and parasitism on nutritional content regulation in host *Tenebrio molitor* L. (Coleoptera: Tenebrionidae). Springerplus 5, 1017. doi: 10.1186/s40064-016-2732-1 27441136 PMC4938838

