# Supplementary material for: Diversity analyses of bacterial symbionts in four Sclerodermus (Hymenoptera: Bethylidae) parasitic wasps, the dominant biological control agents of wood-boring beetles in China
Source: Front Cell Infect Microbiol. 2024 Jul 25;14:1439476. doi: 10.3389/fcimb.2024.1439476 (PMC11306144; doi:10.3389/fcimb.2024.1439476)
Supplement: Supplementary file 1 [file Presentation_1.pptx]

## Slide 1
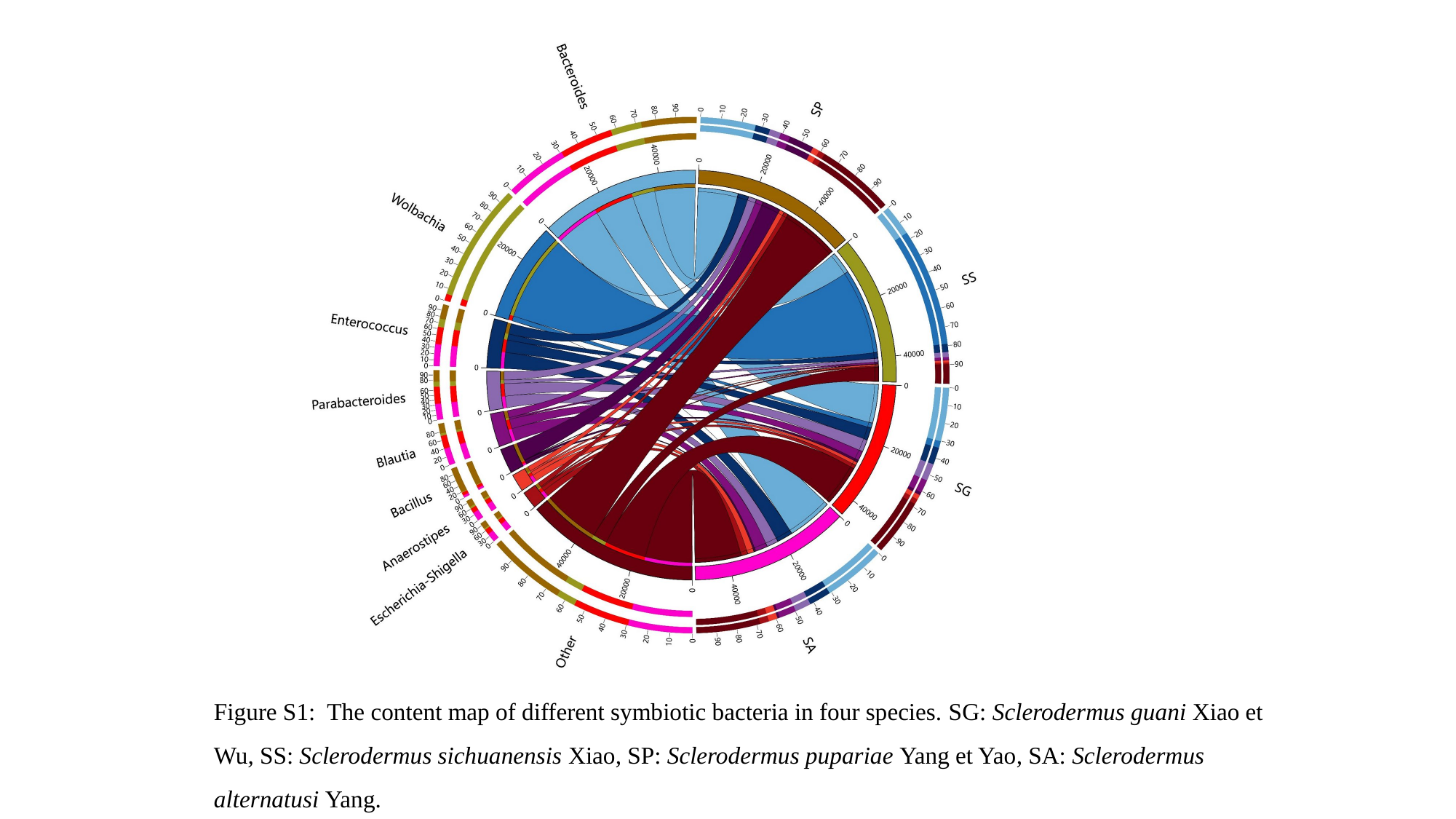

Figure S1: The content map of different symbiotic bacteria in four species. SG: Sclerodermus guani Xiao et Wu, SS: Sclerodermus sichuanensis Xiao, SP: Sclerodermus pupariae Yang et Yao, SA: Sclerodermus alternatusi Yang.
